# Online Intervention as Strategy to Reach Men Who Have Sex With Other Men and Who Use Substances in a Sexual Context. Development of the MONBUZZ.ca Project

**DOI:** 10.3389/fpsyt.2019.00183

**Published:** 2019-04-09

**Authors:** Jorge Flores-Aranda, Mathieu Goyette, Catherine Larose-Osterrath

**Affiliations:** ^1^Centre Intégré Universitaire de Santé et de Services Sociaux, Centre-Sud-de-l'Île-de-Montréal, Institut Universitaire sur les Dépendances, Montreal, QC, Canada; ^2^Département des Sciences de la Santé Communautaire, Université de Sherbrooke, Sherbrooke, QC, Canada; ^3^Département de Psychologie, Université du Québec à Montréal, Montreal, QC, Canada

**Keywords:** sexualized substance use, MSM (men who have sex with men), online interventions, outreach, community-based research

## Abstract

Men who have sex with men (MSM) use more psychoactive substances and a greater variety of them compared to their heterosexual peers. In this population, substance use is particularly characterized by polydrug use, binge, and sexualized substance use. MSM who use substances do not recognize themselves in public health messages targeting substance users. In addition, they recognize their problematic substance use later than heterosexuals and, as a result, they use addiction services later in their addiction trajectories. When accessing addiction services, the links between drug use and sexual life are rarely considered. Because of this profile, online interventions are a promising way to reach this hard-to-reach population. Currently available online interventions targeting MSM address the topics of substance use and sexual life separately. To deal with this situation, our team wanted to develop an online intervention platform for MSM who use substances in a sexual context. Given that online addiction interventions do not address sex and that MSM drug use is highly related to sexual activity, we first explored the literature related to online interventions targeting MSM and HIV risk behaviors, as well as online interventions targeting general population in order to: (1) identify relevant (or personalized) intervention methods; (2) describe the approaches used; and (3) describe their effects. Second, we turned to the literature to develop the MONBUZZ.ca project in collaboration with community organizations. The results of the narrative review provided a critical portrait of online interventions for MSM and guided the development process of MONBUZZ.ca. We discuss issues of co-development of a research and brief intervention tool based on promising practices as well as challenges of its implementation and evaluation.

## Introduction

Men who havesex with men (MSM) tend to use more psychoactive substances than do men who do not have sex with men (1–3). Among MSM who use drugs, some use these substances mostly in a sexual context (4, 5) and the phenomenon of “chemsex” has been increasingly documented. “Chemsex” refers to the use of substances such as methamphetamines (especially in North America and Australia), mephedrone (especially in Europe), GHB/GBL and ketamine to extend, intensify or diversify sexual activities (1, 6, 7). Other authors characterize chemsex by the combined use of these substances and other products such as erectile dysfunction drugs and certain substances, sometimes sold legally, such amyl nitrites (*poppers*) (8). More broadly, Hickson (9) defines chemsex as a social and cultural phenomenon that involves voluntary risk taking, powerful emotions and strong sensations within the context of dating apps and biomedicalized sexuality (i.e., use of HIV pre-exposure prophylaxis, or PrEP). There is no consensus around the definitions of “chemsex.” However, the definitions presented above suggest that type of substances varies depending on cultural and geographical context. These definitions also incorporate intent (or motivation) to use these substances to enhance different aspects of the sexual experience. In this article we will use “substance use in a sexual context” to refer to use of any psychoactive substances, including alcohol, in a sexual context, regardless of the intention or function sought by the users. Substance use in a sexual context is sometimes associated with health-risk sexual behaviors (1, 6, 7). In addition, condomless sex is also common among MSM using substances in a sexual context (8, 10), as is injection drug use and sharing of injection paraphernalia (10–12). Therefore, substance use in a sexual context among MSM represents a major public health concern (10), which could be translated into public health activities that take into account the context in which substance use takes place (9).

Some MSM with problematic substance use do not receive services for this problem (3) for several reasons. First, like many other substance users, some MSM with problematic substance use are unaware of the problem. In turn, their lack of awareness explains why prevention and treatment messages targeting substance users do not resonate with this audience (3, 13). Second, problematic substance use is not always detected by health care professionals who work either in general services or specialized sexual health services (3). Therefore, their substance use profiles are sometimes more severe once they enter into services (11). Third, fear (which can be well-founded) of health care workers' negative attitudes toward LGBTQ+ people and a theoretical lack of addiction services adapted to their needs decrease MSM's use of addiction services (14). There is a consensus about the magnitude and severity of substance use among some MSM and about the fact that they are a difficult population to reach (15). Therefore, specific actions are necessary to reach this population, whose use of services is limited. In this context, online interventions represent a promising strategy to reach and serve this population (16).

Online interventions provide care exclusively over a computer or mobile device and are sometimes combined with traditional services (17). These interventions convey general information or information tailored to each user's profile, and also include virtual discussion spaces where individuals can talk with a health care professional via synchronous or asynchronous interfaces (17). These platforms can be in the form of educational modules on dedicated web platforms, text messages, live chat sessions, discussion forums, social media interventions or ecological momentary interventions (17, 18). Online interventions have a number of benefits. For example, not having to talk to someone in person reduces feelings of stigma or discomfort that users of health care services may experience with health care professionals (19, 20). These interventions can also eliminate the financial, time, and geographical barriers of traditional services, thereby improving access to hard-to-reach populations (21). Finally, gay, bisexual and other MSM, like many other populations including young people, have quickly adopted the Internet to socialize and get health information, and are willing to participate in Internet research projects (22). Therefore, online interventions represent an outreach opportunity to interact with these populations in their daily lives.

Our team wanted to develop an online intervention tool for MSM who use substances in a sexual context. It was important to integrate the experiences and best practices from the scientific literature to provide the most rigorous intervention possible and overcome challenges identified by other authors and by the stakeholders collaborating in the project. Given that most online addiction interventions do not address sex and MSM substance use in a sexual context is a concern, we first explored the literature related to online interventions targeting MSM and HIV risk behaviors, as well as online interventions that target substance users (regardless of sexual orientation or sex with same sex partners). Our aim was to (1) identify relevant (or personalized) intervention methods; (2) describe the approaches used; and (3) describe the effects of those methods. Second, we turned to the literature to develop the MONBUZZ.ca project in collaboration with community organizations. The project co-development will be described and discussed in the second part of this article, as well as the use made of scientific documentation throughout the development process of MONBUZZ.ca.

## Methodology

### For the Narrative Literature Review

To meet the above-mentioned goals, we explored three major concepts: online interventions; sexual health and prevention of HIV and other sexually-transmitted and blood-borne infections (STBBI); and substance use. Multiple keywords were defined for each concept and combined with Boolean operators (for example, substance use, sexualized substance use, risk behaviors, online interventions, web-based interventions). These themes and keywords were searched in the following databases: MEDLINE with Full Text; CINAHL Plus with Full Text; ERIC; FRANCIS; PASCAL; PsycARTICLES; PsycCRITIQUES; PsycEXTRA; Psychology and Behavioral Sciences Collection; PsycINFO; Social Work Abstracts; and SocINDEX.

Since the goal of the review was to further our reflection on how to develop the MONBUZZ.ca platform, we decided to focus on studies about the effectiveness of similar interventions. Our selection criteria for this review consisted of articles about randomized controlled studies of interventions aimed at changing substance use habits or at-risk sexual behaviors. The article also had to be published in English or French and pertain to MSM or adult gay/bisexual men. We excluded articles that did not address the impact of interventions as well as reviews. Data extraction was performed by two research professionals. Given the diverse goals of the interventions and varied study populations and contexts, we performed a narrative analysis. A narrative analysis allows inclusion of documents with a wide variety of methodological terms. This analysis did not allow us to draw conclusions based on the level of evidence in the selected studies; however it was particularly useful for identifying the main themes surrounding a subject, in this case online interventions targeting MSM (23).

## Results

### Study Selection

For the narrative review, an initial literature search of online interventions and substance use yielded 2,644 results. The literature search on the theme of online interventions to address the sexual health of MSM yielded 80 results, while the one on sexual health, substance use and online interventions targeting MSM generated only 8 results. Given the scarcity of studies specifically focusing on our theme of interest, we decided to consider (1) substance use online interventions targeting heterosexuals (*n* = 601); and (2) sexual health online interventions targeting MSM, specifically the prevention of HIV and other STBBIs (*n* = 80). After assessing the relevance of 681 titles and abstracts according to the selection criteria, we selected 26 studies.

### Description of Studies Selected for the Review

Twenty-six articles were selected for the review. The online interventions described in these studies aimed to increase knowledge about the prevention of HIV and other STBBI as well as to decrease at-risk sexual behavior (24–31). Some online interventions aimed to increase the use of HIV or STBBI testing among adult MSM (32, 33) or young adult MSM (34) who live in urban or rural settings (24). The interventions of the reviewed studies are based on different theoretical approaches, such as the integrated behavioral model (34), health belief model (32), information motivation behavioral skills model (24, 25, 29, 31), social learning theory (26), STD-related cognitive approach and fear appeal approach (28), and the sexual health model approach to HIV prevention (30).

In terms of intervention approaches, most were carried out with multiple modules (24, 25, 29, 31) that included videos or multimedia presentations (25, 29–31). In some studies, different types of video (informative, documentary or dramatic) were used for the intervention (26, 28, 32). In some cases, the interventions were adapted to the participants' profiles (27, 31) or how they identify themselves with regard to their sexual orientation or gender, e.g., they could choose a character in a story based on the sexual identity they listed to take part in the intervention (32). The participants received personalized feedback based on their profile or their answers to the questions asked (27, 31, 34). In one study, the intervention was done via e-mail (27). In other cases, it was done via a closed group on a social network, with discussions between moderators and participants (33).

### Effects of Interventions on Testing for HIV and Other STBBI

Bauermeister et al. (34) conducted a randomized controlled trial to assess the effectiveness of a pilot project to promote the testing of HIV and other STBBI among young MSM (13–25 years old). The eligible participants were assigned to either an experimental intervention (a personalized website called Get Connected!) or a controlled intervention (a website that allowed people to locate screening services within their geographic reach). All participants completed a baseline questionnaire that was used, among other things, to personalize the navigation on the web site. Those in the experimental group had access to four webpages: the first page presented an information table on different STBBIs; the second assessed participants' motivations, strengths and values around STBBI screening; the third evaluated barriers to screening; and the fourth offered a list of resources, which was identical to that received by participants in the control group. Finally, participants were asked about the feasibility and acceptability of the intervention (single session intervention). Overall, 130 young MSM participated in the study and 104 responded to the 1 month follow-up (brief survey measuring the primary outcomes). Compared to their peers in the control intervention, the participants exposed to the experimental intervention were significantly more likely to ask their sexual partners to get tested for HIV (2.27 vs. 1.75; *t* = 2.59; *p* < 0.05) and to get tested for a STBBI (2.22 vs. 1.81; *t* = 1.95; *p* < 0.05). They were also significantly more likely to educate other people about HIV and STBBI using the information they received through the intervention (2.72 vs. 2.14; *t* = 2.63; *p* < 0.01).

An increase in testing among MSM who do not identify as gay was also observed in the randomized controlled trial of Blas et al. (32). The goal of this study was to compare the impact of a video intervention using a motivational approach (experimental group) and a standard public health message in a text format (control group) on HIV testing. The video intervention was tailored to three types of MSM: those who self-identify as gay, those who do not self-identify as gay, and trans people. The tailored videos lasted 5 min; participants were exposed to one video, depending on their profile (single session intervention). The article focuses on two sub-groups (MSM who self-identify as gay and those who do not self-identify as gay). A total of 239 participants were assigned to the experimental intervention: 142 in the group of men identifying as gay and 97 in the group who did not identify as gay. The control group had 220 subjects that included 130 men who identified as gay and 90 who did not identify as gay. Compared to the participants in the control group, the participants who were part of the experimental intervention and who did not identify as gay were more inclined to report an intention to get tested for HIV in the 30 days following the intervention (*RR* = 2.77; 95% CI: 1.42–5.39). This same group was also more inclined to make an appointment over the Internet to get tested for HIV (*RR* = 1.48; 95% CI: 1.13–1.95).

In a more targeted way, the study of Young et al. (33) aimed to determine whether an intervention through closed Facebook groups would increase HIV testing with a self-testing kit among African American or Latin American MSM who live in Los Angeles. The main outcomes measured were the actions of ordering a self-testing kit, returning the kit, and following up to get the results. This study consisted of two experimental groups and two control groups (all groups made up of African American or Latin American MSM). For 12 weeks, the experimental groups were exposed to information about HIV, whereas the control groups received general health information. Participants were assigned either to one of the experimental groups or one of the control groups. Each group had moderators who were randomly assigned to one of the 4 groups. Participants were instructed to use Facebook as they normally would. They were not obliged to respond to moderator messages or other members of the closed group. In addition, they could control the information they wanted to share with others in the group. A group operating guide was also given to them. Moderators could start discussions and remind participants to take part in the discussion weekly. A total of 112 MSM participated in the study: 57 were assigned to the experimental intervention and 55 to the control group. Of these, 53 participants from the control group completed the follow-up, while 52 from the experimental group did it. Every 4 weeks, the participants received messages telling them that they could order a free HIV self-testing kit that they could use at home. The results showed that more subjects assigned to the experimental intervention ordered HIV self-testing kits compared to those in the control group [44 vs. 20%, mean difference, 24% points (95% CI, 8–41% points)]: 25 participants in the experimental group ordered self-testing kits; among them, 9 returned the kits and 8 followed up to get the results. In the control group, 11 participants ordered self-testing kits; among them, 2 returned the kits and none followed up to get the results.

### Impact on HIV and Other STBBI Sexual Risk Behaviors

Carpenter et al. (25) conducted a randomized controlled trial to evaluate the effectiveness of an online intervention with regard to the at-risk sexual behavior of MSM whose HIV status was negative or unknown. All participants were asked to complete a baseline questionnaire (lasting about 25 min) before being randomized to the experimental or control group. A total of 81 MSM completed the experimental intervention and 73 completed the control intervention. Of this number, 59 (experimental group) and 53 (control group) completed the 3 months follow-up. The experimental intervention included seven interactive modules lasting about 20 min that could be completed within 1 week. This intervention aimed to reduce the risks associated with HIV and other STBBI, to improve the participants' abilities to engage in safer behavior and increase their motivation to change their behavior. The control intervention consisted of a stress reduction training program called eTranquility. Controlling for general time effects, participants in the experimental group tended to show a decrease in self-reported instances of unprotected sexual intercourse with at-risk partners for the following practices: anal intercourse in general (*F* = 7.59; *df* = 1.101; *p* = 0.007; η^2^ = 0.070), insertive anal intercourse (*F* = 7.24; *df* = 1.101; *p* = 0.008; η^2^ = 0.067); insertive oral intercourse (*F* = 7.45; *df* = 1.101; *p* = 0.007; η^2^ = 0.069); and receptive oral intercourse (*F* = 8.45; *df* = 1.101; *p* = 0.004; η^2^ = 0.077). However, there was no decrease for receptive anal intercourse (*F* = 4.79; *df* = 1.101; *p* = 0.248; η^2^ = 0.013). Relatively similar results in terms of decreased at-risk practices were obtained in the study by Bauermeister et al. (34), the methodology of which is described above. The authors observed that, 30 days after the intervention, participants in the experimental and the control groups had significantly increased safer sex practices. They reported fewer sexual partners (1.84 vs. 1.39; *t* = 2.26; *p* < 0.05), less receptive anal intercourse (0.80 vs. 0.56; *t* = 2.43; *p* < 0.05), less unprotected receptive anal intercourse (0.46 vs. 0.29; *t* = 2.90; *p* < 0.05) and less insertive anal intercourse (0.72 vs. 0.55; *t* = 1.99; *p* < 0.05).

The goal of the randomized controlled trial by Hirshfield et al. (26) was to evaluate the impact on HIV of five different interventions, including a 9 min dramatic video, a 5 min documentary video, both the dramatic and documentary videos (broadcast randomly), and a prevention webpage (experimental group interventions), in comparison with web links to HIV information and prevention resources (control group intervention). All participants could view the information only once. A total of 3,092 MSM participated in the study, and 1,631 participants completed the 60-days follow-up. After 60 days, a decrease in unprotected anal intercourse was observed among HIV-negative participants who were exposed to different videos (*OR* = 0.70; 95% CI: 0.54–0.91) as well as among participants exposed to the information and prevention pages (*OR* = 0.43; 95% CI: 0.25–0.72). The HIV-negative participants who watched the videos reported a decrease in unprotected anal intercourse (*OR* = 0.38; 95% CI: 0.20–0.67) and in unprotected anal intercourse with a casual and serodiscordant partner (*OR* = 0.53; 95% CI: 0.28–0.96), compared to participants exposed to other interventions. The participants exposed to one of the videos were more likely than those in the control group to disclose their HIV status to their sexual partner (*OR* = 1.51; 95% CI: 1.16–1.98) as well as to ask their partner about their status and disclose their own (*OR* = 1.32; 95% CI: 1.01–1.74).

Regarding the effects of some approaches on changes in at-risk sexual behavior, Lau et al. (28) conducted a three-arm randomized controlled trial to assess the effectiveness of an online intervention based on the following: Sexually transmitted diseases (STD)-related cognitive approach (arm 1) and STD-related cognitive approach plus fear appeal imagery approach (arm 2), compared to a control intervention (informative webpage about HIV) (arm 3). In the experimental arms, participants were exposed to three videos: two 5 min videos based on an STD-related cognitive approach, and a 10 min video based on fear appeal approach (experimental interventions), in comparison with an informative intervention about HIV (control intervention). In total, 396 men participated in the study, 133 were assigned to the STD-related cognitive approach (94 completed the study), 133 to the STD-related cognitive approach and fear appeal approach (109 completed the study), and 136 to the control group (102 completed the study). The participants were followed at 1 and 3 months after the intervention. The study found no significant relationship between the level of fear induced by the interventions and the prevalence of unprotected anal intercourse. However, significant differences were observed within some groups. In comparison to baseline, results after 3 months showed a significant decrease in unprotected anal intercourse with regular partners and with all types of partners among participants in the three study arms.

### Maintaining Behavior Changes Over Time

The study by Mustanski et al. (29) assessed the feasibility, acceptability and preliminary efficacy of an online intervention called *Keep It Up!*. The intervention included 7 modules and a booster session which could be completed within 24 h. Each module lasted about 2 h. The intervention control also consisted of seven modules (which were shorter than those in the experimental intervention and did not include interactive tools). The participants had follow-up assessments at 6 and 12 weeks after the intervention. A total of 102 participants were included in the study −50 assigned to the experimental group and 52 to the control group. Among the 50 subjects assigned to the experimental group, 44 completed the 6 weeks follow-up and 41 completed the 12 weeks follow-up. Of the participants in the control group, 50 completed the 6 weeks follow-up and 49 completed the 12 weeks follow-up. After 12 weeks, participants in the experimental group had a lower rate of unprotected anal intercourse compared to participants in the control group (*RR* = 0.56; *p* < 0.05). Intervention acceptability was measured immediately after the intervention and a mean acceptability score was calculated. The authors reported a good level of acceptability for participants in the experimental group (*M* = 5.29, *SD* = 0.73) and for those in the control group (*M* = 5.31, *SD* = 0.67).

Rosser et al. (30) observed significant changes in at-risk behavior in their study; however, these changes were not maintained over time. The goal of their randomized controlled trial was to test an online interactive intervention to prevent HIV among MSM. The intervention consisted of modules that users could complete within a 7 days. The modules included interactive gamified tools along with video segments and animations. The article does not provide any information about the number of modules nor about the lasting effects of each module. For the control group, the participants were put on a waitlist. All participants were invited to fill out follow-up surveys at 3, 6, 9, and 12 months. Overall, 650 MSM participated in the study. Of this number, 337 were assigned to the experimental group and 313 to the control group. Retention rates at 12 months were 82% (experimental group) and 89% (control group). The results contrasting the first (after the baseline) and the last measurement time showed at the 3 months follow-up, a decrease of 15.6% in condomless anal intercourse among participants in the experimental group (95% CI: 0.704–1.013; *p* = 0.0068). No significant difference was observed at the 12 months follow-up.

### Improved Knowledge About HIV and Condom Use

The randomized controlled trial of Schonnesson et al. (31) assessed the effectiveness of an online intervention (SMART) that aimed to reduce HIV risk behavior among Swedish MSM. The intervention was divided into three interactive modules with personalized feedback based on the participants' responses. Each module lasted ~20 min and was divided into 2 sessions. The participants had 48 h to complete each session and had to wait 24 h before completing the next module. Follow-up was done after 30 days. The participants were assigned to either the experimental intervention (SMART) or a waitlist (control group). Overall, 112 MSM participated in the study: 54 were assigned to the intervention and 58 to the control group. The 1 month follow-up retention rate was 43% for the experimental group and 61% for the control arm. The participants in the experimental group improved their knowledge about HIV (*OR* = 2.02; 95% CI: 1.18–3.46; *p* < 0.01), and they also had a greater belief that condom use was an act of responsibility (*OR* = 3.28; 95% CI: 1.07–10.06; *p* < 0.04). Compared to participants in the control group, participants in the experimental group were more likely to use a condom with every new partner all the time (*OR* = 4.01; 95% CI: 1.13–14.20; *p* < 0.03). They were also more likely to experience increased personal effectiveness when using condoms in challenging situations (*OR* = 5.19; 95% CI: 1.31–20.59; *p* < 0.02).

Lau et al. (27) conducted a randomized controlled trial to evaluate the effectiveness of an online intervention that included periodic information on HIV, the monitoring of behavior with interactive feedback, as well as peer counseling. The experimental intervention consisted of emails sent bi-weekly over 6 months with graphics adapted to the target population about modes of HIV transmission, correct condom use, HIV testing, emotional relationships, as well as the links between substance use and sexual activity. Each month, the participants had to fill out a questionnaire about their HIV sexual risk behavior in the past 30 days. A total of 477 men participated in the study, of which 140 participants from the experimental group and 140 from the control group completed the follow-up at 6 months. The participants were men aged 18 years and over who had reported engaging in oral or anal sex with a man in the previous 6 months and who regularly use the Internet. The control intervention consisted of sending educational materials on the topic. No significant difference was observed between the groups at baseline or at the 6 months follow-up.

Just one study addressed the use of substances and at-risk sexual behavior (35); however, this study only focused on a single substance (methamphetamine). Although it was not a randomized trial, we included it in the literature review because of its relevance to the project we were developing. This study evaluated a pilot intervention with 52 MSM who had reported having unprotected anal sex and using methamphetamine in the previous 2 months. Interventions over text message were developed based on the social support theory, social cognitive theory, and health belief model. Predetermined text messages personalized to the participants' profiles were sent out daily for 2 weeks. Follow-up was done 2 months later. The results showed that exposure to messages based on the health belief model and the social cognitive theory significantly reduced the self-reported use of methamphetamine. Also, the messages based on social cognitive theory significantly reduced the number of occasions of unprotected anal intercourse with a serodiscordant partner as well as transactional sex.

### Online Interventions to Change Substance Use in the General Population

Campbell et al. (20) conducted a randomized controlled trial to evaluate the effectiveness of an online intervention called the Therapeutic Education System (TES) in addition to in-person interventions. Participants were assigned to either the usual intervention (control group) or to the usual intervention combined with TES (experimental group). The two interventions were conducted over 12 weeks, with follow ups at 3 and 6 months. Urine tests were performed at follow up visits. The TES included 62 interactive modules lasting 20 to 30 min and based on contingency management. Overall, 255 people were assigned to the experimental intervention and 252 to the control group. Participants in the experimental group completed an average of 36.6 (SD = 18.1) of the 62 TES modules. Participants in the experimental group had higher abstinence rates than those assigned to the control group (*OR* = 1.62; 95% CI: 1.12–2.53; *p* = 0.010). The hazard ratio values showed that the former were also less likely to abandon treatment (*HR* = 0.72; 95% CI: 0.57–0.92; *p* = 0.010).

Lewis et al. (36) conducted a randomized controlled trial to evaluate the effectiveness of an intervention based on personalized normative feedback about alcohol-related risky sexual behavior (RSB). After completing the baseline in approximately 20 min, participants answered a 30 min questionnaire (single session intervention). They were then asked to complete a 50 min questionnaire 3 and 6 months after completing the baseline. Participants could be assigned to one of four online interventions developed using social learning theory. Three interventions were experimental (alcohol-only, alcohol-related RSB-only, combined alcohol and alcohol-related RSB) and one a control intervention. Participants were university students aged 18 to 25 years attending a public university in the United States. A total of 480 students participated in the study: 119 were assigned to the alcohol-only intervention, 121 to the alcohol-related RSB-only intervention, 119 to the combined alcohol and alcohol-related RSB intervention, and 121 to the control group. The 6 months retention rate was 85%. Compared to the participants in the other groups, those assigned to the alcohol-only and alcohol-related RSB-only interventions reduced their frequency of drinking alcohol by between 10 and 20% at 3 and 6 months after the intervention. Three months after the intervention, the participants in these same groups had decreased their frequency of drinking alcohol before sexual intercourse.

The goal of the randomized controlled trial conducted by Sinadinovic et al. (37) was to assess the efficacy of two online interventions (experimental arm): a brief personalized normative feedback intervention (eScreen.se) and a self-help intervention based on the principles of cognitive-behavioral treatment (Alkoholhjalpen.se) (experimental interventions). In one session, sScree.se measured consumption of alcohol and other substances, the place of substance use in the lives of respondents and their readiness to change. A personalized assessment based on the answers and references to appropriate services were then offered. Alkoholhjalpen.se consisted of 18 modules with interactive activities that participants completed as they wished. The platform also provided opportunity for discussion in an open forum with other participants. Participants could log in to both websites with personalized usernames and passwords, and use the websites as they wanted with no time limit. The control intervention was a web-based screening tool. The target outcome of the interventions was to reduce problematic alcohol use. The participants were followed at 3, 6, and 12 months. Overall, 633 people participated in the study: 211 were assigned to eScreen.se, 212 to Alkoholhjalpen.Se, and 210 to the control group. The 12 months retention rates were 59.2, 54.3, and 52.4%, respectively. After taking attrition into account, the average scores from the tools used to measure alcohol use decreased significantly for participants in all intervention groups (experimental and control) at 3 months. These scores remained stable but not statistically significant at 6 and 12 months.

The eScreen.se project was the focus of a randomized controlled trial by the same team (38). The purpose of this study was to assess the effects of this Internet-based screening and brief intervention site (eScreen.se) compared to web-based screening-tool only control group. Measurements were taken at 3 and 6 months after the intervention. In total, 202 people participated in the study, 101 of whom were assigned to the experimental group and 101 of whom were assigned to the control group. The attrition rates in the experimental component were 67.3% at the 3 months follow-up and 73.3% at 6 months follow-up. For the control group, these rates were 67.3% at 3 months and 65.4% at 6 months. The average scores on instruments measuring substance use, including alcohol, decreased significantly in the two groups (experimental and control) at the 3 months follow-up. However, the significant decrease was observed at the 6 months follow-up only for the experimental group.

Tait et al. (21) randomized controlled trial assessed the effectiveness of the “Breakingtheice” online intervention on the consumption of amphetamine-type stimulants. The control group was placed on a waitlist and could only access the intervention 6 months later. The participants could log on to the intervention site with a username and password. A total of 160 people participated in the study, 81 of whom were assigned to the experimental intervention and 79 of whom were assigned to the control intervention. Retention rates at 6 months follow-up were 47% for the experimental group and 52% for the control group. The intervention consisted of three modules based on cognitive behavioral theory and motivational enhancement theory. Participants had to complete each module within a week and could not advance to the next module before finishing the previous one. Compared to those in the control group, the participants in the experimental group were significantly more likely to seek help for their substance use (*RR* = 2.16; 95% CI: 1.14–4.10) or have the intention to seek help (*RR* = 1.17; 95% CI: 1.05–1.31). Participants in the intervention group were more likely than those in the control group to move to the action stage of change (*OR* = 4.13; 95% CI: 1.03–16.58).

Bewick et al. (39) conducted a randomized controlled trial to evaluate the effectiveness of an intervention that gave subjects the chance to have their alcohol use evaluated (with the CAGE questionnaire) and get personalized online feedback. This feedback addressed their degree of alcohol use, the percentage of students who reported drinking less alcohol than the participant, and general information. The participants entered a secure code to see their profile via the study webpage. This webpage was available for 12 weeks and participants could visit it as they wanted. The control intervention only provided an assessment of alcohol use. A total of 539 participants were assigned to the experimental group and 536 to the control group. Of this number, 179 participants in the control group and 138 in the experimental group completed the 12 weeks follow-up. The participants assigned to the experimental group reported a lower average alcohol consumption per occasion compared to the control group (*F* = 5.74; *df* = 1,313; *p* = 0.02).

Bock et al. (40) conducted a randomized controlled trial to assess the feasibility, acceptability and preliminary efficacy of a program to reduce alcohol use via text message (Text Message Alcohol Program: TMAP). This program was compared to text messages on motivation in general (not related to alcohol use). The participants were community college students, and they were followed for 6 and 12 weeks after the baseline assessment. A total of six scheduled messages per week were sent out for 6 weeks. The participants were then asked to rate the text messages received on a 10-point scale. The messages in the experimental intervention included information on alcohol, strategies to limit alcohol use and related risks, as well as motivational messages. Overall, 31 people were assigned to the experimental intervention and 29 to the control group, and 93.3% of all participants completed follow-up at week 6, while 88.3% did so at week 12. At week 12, proportionately more participants in the experimental group compared to their peers in the control group (48.4 and 34.5% respectively) reported less than one episode of heavy drinking in the previous 2 weeks (*OR* = 1.78; 95% CI: 0.63–5.04). Compared to participants in the control intervention, those assigned to the experimental intervention were significantly less likely to have experienced negative consequences from their alcohol use (*OR* = 4.77; 95% CI: 1.17–19.40).

Copeland et al. (41) randomized controlled trial assessed the short-term efficacy of two interventions that addressed problematic cannabis use: an intervention combined with brief feedback and an intervention combined with extended feedback. These interventions were based on the motivational approach and were part of the website Grassessment: Evaluate Your Use of Cannabis. Overall, 156 participants were assigned to the intervention with brief feedback and 131 to the intervention with extended feedback, and 68% of participants completed the 1 month follow-up. Participants in both groups significantly reduced the median number of days of cannabis use in the previous month as well as the amount used in the same period. Only participants in the brief feedback group showed a significant decrease in the severity of their dependence between baseline and1 month follow-up.

The randomized clinical trial of Gustafson et al. (42) assessed whether patients leaving treatment for alcohol problems and who had access, in addition to the usual services, to an application that provided support for their recovery (experimental group) would report fewer risky drinking days compared to those with no access to the application and who received the usual post-treatment services (control group). The application was called Addiction-Comprehensive Health Enhancement Support System (A-CHESS). This app had static and interactive content and participants could use it for 8 months. Through the project, participants with access to the application received a smartphone loaded with the application as well as phone service and a data plan. The participants were followed at 4, 8, and 12 months after baseline. A total of 170 people were assigned to the experimental intervention and 179 to the control group. The 12 months retention rates were 77.7% for the experimental group and 77.6% for the control group. At 12 months, participants in the experimental group reported significantly fewer risky drinking days compared to those in the control group. Again at 12 months, participants assigned to the experimental intervention were also significantly more likely to report more days of abstinence in the previous month than those assigned to the control group (*OR* = 1.94; 95% CI: 1.14–3.31).

McCambridge et al. (43) conducted a three-arm randomized controlled trial to evaluate the effectiveness of a brief online intervention to change alcohol consumption habits among university students. Participants were assigned to three possible interventions: alcohol use evaluation and feedback (arm 1); alcohol use evaluation only (arm 2); and, information web site about alcohol (arm 3). Overall, 4,969 people were assigned to arm 1, 4,969 to arm 2, and 4,972 to arm 3. Only 25.1% of all participants completed the 3 months follow-up after baseline. Significantly, participants assigned to arm 3 reported 3.7% more at-risk alcohol consumption compared to participants in arm 1. They were also significantly more likely to report episodes of heavy drinking.

Palfai et al. (44) conducted a randomized controlled trial to examine the effectiveness of an email with a link to a web-based screening and brief intervention for alcohol use? evaluation and prevention. The experimental group consisted of participants who received feedback on their alcohol use (*n* = 890), while the control group included participants who received general feedback on their health (*n* = 446). The intervention lasted about 15 min. The participants were first-year university students in the United States. Follow-up was conducted at 5 months after baseline. The retention rate of participants who had agreed to be contacted for follow-up was 62%. The only significant results were observed among participants who had reported not drinking alcohol at baseline. Participants who did not drink alcohol at baseline and who were assigned to the experimental group were less likely to have consumed alcohol at the 5 months follow-up compared to participants in the control group who also reported not drinking alcohol at baseline (*OR* = 0.50; 95% CI: 0.26–0.98).

## Discussion

### Main Findings From the Literature and Development of MONBUZZ.ca

Randomized controlled trials conducted on non-LGBTQ+ population have demonstrated the effectiveness of interventions to decrease the use of alcohol (20, 36, 37) or other substances (21, 38). Other studies have supported the effectiveness of online interventions to reduce unprotected sex among MSM (25, 29, 30). Just one study addressed the use of substances and at-risk sexual behavior (35); however, this study only focused on a single substance (methamphetamine).

The relevance of using Internet to reach MSM and offer them online interventions regarding HIV or other STBBI has shown promise (45, 46), to the extent that the World Health Organization (47) has recommended using the Internet to reach MSM with at-risk profiles. However, we still know little about interventions that can address both substance use and health-risk sexual behaviors related to substance use. We do know that substance use among MSM is associated with sexual activities and sometimes to health-related sexual risk behaviors (1, 7). Studies that have assessed the effectiveness of online interventions with substance users did not address sexual aspects and did not target MSM (20, 21, 37). On the other hand, studies of online interventions that do target MSM focus above all on at-risk sexual behavior and do not address substance use.

The reviewed literature shows that online interventions can change some risk behaviors among MSM in different settings (32, 33). However, it can be seen that in interventions with multiple follow-up times, attrition rates can vary significantly, ranging from 75% (39, 43) to 4% (33). The extent of these rates is greater than those observed by Vandelanotte et al. (48) regarding attrition rates in web-based health interventions, which ranged from 10 to 50% after 3 months. In addition to methodological differences, the studies consulted showed significant variations regarding the number of follow-up times and the duration of these follow-up interventions. Therefore, it is difficult to make assumptions about the reasons for attrition rates. However, given our observations, we consider attrition prevention as an important issue. We also see that interventions with modules have good results in terms of behavior change but that between-module dropout risks must be considered (24, 25, 31).

This literature review indicates that longitudinal interventions are often module-based. However, the number of modules and the duration of each module vary. Nevertheless, this kind of intervention seems interesting in terms of effects but seems less adapted to a particular population, which results in a high dropout rate. The literature also shows that interventions taking place in a single session and offering evaluation and individualized feedback limit drop-out during intervention while allowing changes of certain risk behaviors (37, 38, 41, 43). This may be a more interesting strategy in some contexts, which can be supplemented with complementary modules, thus providing a level of service tailored to the needs of a targeted population.

The interventions that we identified intended to change behavior but did not establish the intervention needs of the participants. Considering that MSM who use substances do not all do so in a problematic way and that MSM who have substance use problems do not always ask for professional help (for various reasons) (3), we believed that developing an intervention that allows participants to assess the degree of their SU would be a necessary first step. Secondly, brief intervention approaches have demonstrated their capacity to modify behaviors among people who use substances (37, 38, 41, 43). Thirdly, we have seen in the literature that interventions based on motivational approaches (25) have demonstrated to be effective at reducing at-risk sexual behavior in MSM. Given that we are interested in MSM whose substance use can range from low to high and that, as a result, their motivation to change their behavior will be highly varied, we believed it necessary to measure the participants' level of motivation to change. This approach appears to be either directly related to effectiveness or a means to promote the commitment necessary to enhance participant retention. Finally, the literature suggests that personalized interventions (31) and interactive activities (25, 29–31) are shown to be effective. Therefore, we decided to develop personalized feedback based on participants' substance use profiles and their level of motivation to change. In addition, the interactivity of MONBUZZ.ca was developed taking into account how to ask the questions and setting up a discussion box. The purpose of these features was to create an interactive interface and offer the opportunity to interact with a community counselor after the intervention.

### Community-University Co-development of the MONBUZZ.ca Project

#### Community Approach

The project was co-developed based on results of the literature review and on the experience and expertise of the community. MONBUZZ.ca was developed using a community research approach in which all members of the team (academic and non-academic) have an equitable partnership throughout all stages of the research process (49, 50). This partnership involves the sharing of expertise as well as a shared responsibility in terms of decision-making, knowledge creation, and improvement of community health through interventions and improvements to public policies (49, 50). The platform was built using a co-construction approach involving stakeholders in addiction and sexual health from the community, public, private, and public health sectors. This allowed us to gain the perspective of stakeholders, researchers and potential users of the platform.

Everyone involved in the project was divided into three committees, which became spaces for discussion and co-construction: (1) The management committee, made up of researchers, managers, and stakeholders of the RÉZO organization, who were directly involved in the project; (2) the development committee, which consisted of potential users of the platform, addiction, and sexual health stakeholders from different environments, and researchers; and (3) the advisory committee, composed mainly of managers and researchers who are subject matter experts and who worked externally on the project. The role of the management committee was to take the required actions to carry out the project. The development committee's role was to support and provide feedback about the implementation of the portal and the interventions. The advisory committee, for its part, guided and assessed the project with regard to the interventions, knowledge transfer activities, and research.

#### Theoretical Approach

MONBUZZ.ca was designed in order to share the best available practices on sexual health, addiction rehabilitation, and online interventions for MSM. The developed intervention model is based on the components and principles of the Screening, Brief Intervention and Referral to Treatment (SBIRT) model (51). This model has demonstrated its effectiveness in reaching the target population, raising awareness, enhancing motivation and reducing the consequences associated with substance use and referring users to services in a variety of contexts and for different populations (51). The SBIRT model components include screening on substance use with validated instruments, personalized feedback based on results, brief intervention, as well as referrals to services. The brief interventions that we have described in this article have been adapted for online format (37, 38, 41, 43). As part of this project, we relied on the literature and the needs of the community to develop the short intervention of MONBUZZ.ca. The Montreal context surrounding substance abuse among MSM and the limited services available led us to develop a platform for self-evaluation of substance use and motivation to obtain personalized feedback (brief intervention that could enhance the motivation for change some health related risk behaviors) and the reference to relevant services according to the profile of the participants. This format appeared relevant at an initial stage of development to offer an adapted intervention in one session, with the possibility of including other interventions in subsequent phases of development. This component seemed appropriate to reach people who were not already receiving services, who may or may not be aware of their problematic substance use, and who are willing to change their substance use habits or health-related sexual risk behaviors.

### Tools to Develop the Web Platform

**Figure 1:**
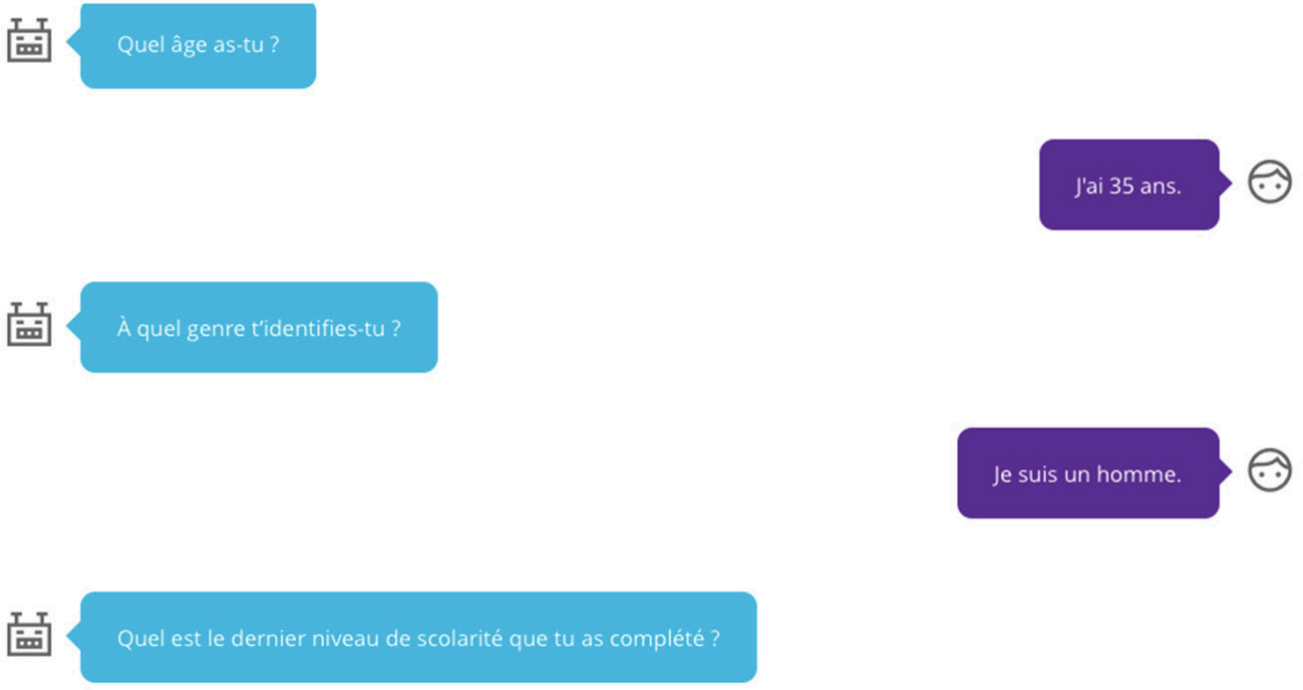
Example of some sociodemographics questions.

The choice of data collection tools was discussed with the management committee and development committee. During this consultative process, the members of the development committee suggested that the language be as accessible as possible. It was decided that the informal second person, or *tu* form, in French would be used in all tools. To help users navigate the platform, questions were asked in the form of a text message conversation (see Figure 1). Two studies cited in this article have successfully used text message interventions (35, 40). We rely on this data as well as the expert opinion (community counselors, clinicians and potential users) for the choice of an interface similar to that of text messages. Again, to help users navigate the platform, the questions were created in the form of scales that can be answered with emoticons or graphical scales. The use of text messaging during the assessment and a chat box after the personalized feedback enhanced interactivity while using well-known formats for smart phones and social media users.

### Participant Questionnaire

For the inclusion criteria, 15 items were developed on the sociodemographic status of the participants, the target population, and how they found out about MONBUZZ.ca. The questionnaire was developed by the management committee to be brief and easy to use so that users would be motivated to finish their evaluation according to the format suggested by the development committee. The indicators were selected based on those recommended in the addiction research (52).

### Questionnaire on Substance Use

The French version of ASSIST 3.0 (53) was used to determine the substances used and to detect a substance use problem. As recommended by the WHO, only the names or some substances were changed to account for regional and cultural differences and to preserve the instrument's validity. This self-reported questionnaire was selected because it has been the focus of many studies that support its reliability, validity and usefulness in different contexts and with diverse populations (53), because it can detect problems with both alcohol and other substances, because it is brief (5–10 min), and because it is consistent with the SBIRT intervention model (54). The results were interpreted using an algorithm to determine, for each substance, use risk level (low, moderate and high) associated with referral to a level of intervention intensity (no intervention, brief intervention, brief intervention followed by intensive treatment), and some possible risks of maintaining regular, moderate or high substance use specific to sexual functioning (54).

### Questionnaire on Motivation to Change Substance Use

An adaptation of the French translation (55) of the *Readiness to Change Questionnaire* (56) was used to determine users' stage of change in terms of their use of alcohol or other substances (precontemplation, contemplation or action) as per the Transtheoretical model of change (57). This self-reported questionnaire was selected because studies of the original version have supported its reliability, validity and usefulness with users of substances, and the questionnaire is shorter than other instruments (58). The results were interpreted using an algorithm to determine the user's stage of change and to adapt the brief intervention to the user.

### Questionnaire on the Influence of Substance Use on Sexual Activity

The Links SU-Sex questionnaire (59) was developed, as there was no instrument that had been studied regarding the influence of substance use on sexual activity, beyond the risk of HIV infection and other STBBI. Its development is part of an iterative process between researchers and the development committee. The developed evaluation had to be relatively short, based on best current knowledge available, address several dimensions of sexuality, show a non-judgmental view of the influence of drug use on sexuality, and be accessible and useful to users. The questionnaire was designed to let participants reflect on the influence of their substance use on their sexual activity, with both the positive and negative aspects of this use, and get feedback on each aspect. In this respect, no overall result was subject to interpretation.

### Steps to Use MONBUZZ.ca

**Figure 2:**
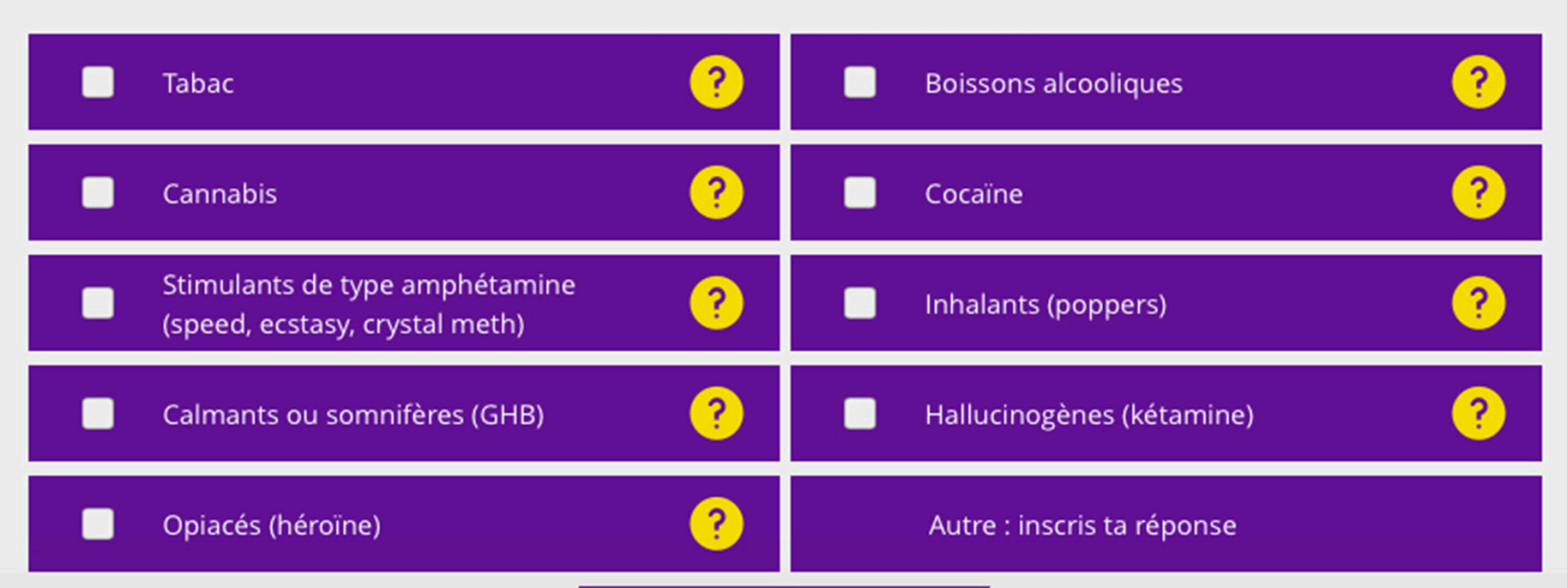
Example of some ASSIT (53) questions.

**Figure 3:**
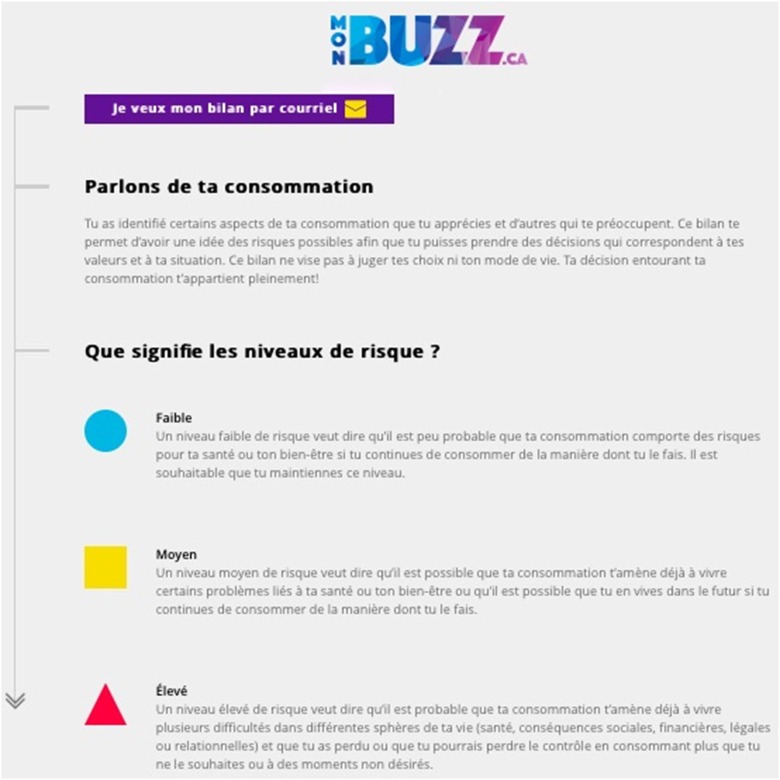
Example of feedback.

The sequence of steps that users take when they get to the MONBUZZ.ca intervention site are as follows: (1) Users arrive at the portal, where the general information is presented (e.g., objectives, content); (2) After consenting to participate in the study, they are asked to fill out a questionnaire on some of their characteristics to ensure that the feedback is tailored to the targeted population (e.g., be an adult man who has had sex with men in the past year); (3) They are invited to complete the screening component (“Screening”) made up of questionnaires on their use of substances (see Figure 2) and the associated consequences, the influence of their use of substances on their sexual activity, as well as their motivation to change their substance use; (4) They are invited to receive automatic and personalized feedback about the above-mentioned elements (see Figure 3), and this feedback is based on best practices in the area of substance use in a sexual context (14, 54, 60–62); (5) When they present with at-risk or problematic use (except for tobacco use) or if they report concerns about the influence of their substance use on their sexual lives, they are asked to take part in a live chat room for 20 to 30 min, immediately following the intervention or at a later time, depending on their preference and the availability of community counselors (“Brief Intervention”); and (6) They are then automatically referred to the best resources for their needs in their region (“Referral to Treatment”). These last two steps are based on the results of some studies cited in this article (37, 38, 41, 43).

### Study Limitations

The studies analyzed in this article report high rates of attrition. *Post-hoc* analyses suggest that these rates vary according to certain sociodemographic characteristics of participants (age, ethnocultural background, HIV status, etc.). This indicates that some populations could benefit from other kinds of online interventions. Moreover, in our sample, 11 studies reported greater proportions of white participants (more than half of the sample) (20, 25, 26, 29, 30, 34, 36, 40–42, 44). Therefore, the results must be interpreted as valid for this population, but not necessarily for minority ethnocultural groups.

In the reviewed literature, there was a heterogeneity in terms of intervention objectives. There were also various needs among the targeted populations. MONBUZZ.ca aimed to reduce substance use risk behaviors, but also to raise awareness about problematic substance use and refer some participants to existing services. This was less in the forefront in reported studies that focused on behavioral changes regarding risk taking. Considering the purpose of MONBUZZ.ca, other elements than efficacy were important to consider.

We did a narrative synthesis of the literature to develop MONBUZZ.ca. As previously mentioned, a narrative analysis allowed us to synthesize data from studies with high variability in methodological terms. Indeed, the studies reviewed focus on the effects of online interventions on various variables associated with both health related risks sexual behaviors and substance use. Given this diversity, it is not possible to calculate the pooled effect of these interventions. Moreover, when the effect size is reported, it varies and is in a low (0.2) to moderate (0.5) level (63). In this sense, we invite readers to interpret with caution some of the reported results.

Finally, another limitation of this study is that it relied solely on the effectiveness of interventions from randomized controlled trials (RCT). Although the findings from these studies are more robust, other components associated with the ability to initiate and maintain participants' engagement in the intervention are important, particularly in online interventions. In addition, in RCT, participants' commitment and follow-up is enhanced by monitoring and financial compensation strategies. Indeed, some authors believe that online interventions must be based on user-centered approaches, encourage engagement and collaboration, and quickly implement and test interventions (64).

## Conclusion

### The Main Challenges of Co-construction

As previously mentioned, MONBUZZ.ca development has followed a co-construction approach involving stakeholders in the field of addiction and sexual health. This allowed us to have the perspective of researchers, addiction counselors and potential users. However, this approach entailed some challenges. Although the goals pursued by academic researchers and community researchers were the same, the visions of the length of the online intervention and its data collection tools could sometimes differ. There has been some negotiation regarding community needs and the need for rigorous clinical and scientific intervention. For example, some community stakeholders and potential users wanted to modify data collection tools to make them shorter and more adapted to the local context. However, for academic researchers, it was essential to use validated tools, which made any modification difficult. In this sense, several discussions were held to evaluate the pros and cons of each position and to decide what was the most appropriate for the project implementation phase. These discussions were inspired by debates about the development of HIV interventions for MSM, described by Otis (50).

The members of the development committee wanted MONBUZZ.ca to be easy to navigate and attractive to users. This aspect of the interventions has not been widely discussed in the reviewed articles but is of great importance. Committee members dismissed from the outset the possibility that the MONBUZZ.ca looks like a web questionnaire. After several discussions between the development committee and the firm that developed the website, it was decided that the questions asked in each of the data collection tools would look like a chat window. For example, each question was asked by a robot in a personalized way. Visually, questions and answers looked like a text exchange. In addition, when questions required answer choices, each choice was represented by an emoji. These choices reflect both the needs of academic researchers (using validated instruments) and the preferences community researchers (ensuring that the platform was attractive to participants).

### Perspectives in Research and Intervention

MONBUZZ.ca was launched in September 2017 and data collection continued until May 2018. Preliminary analyzes show that 152 MSM completed their assessments (out of 237 men who started to navigate the site, which represents 64.1%). The issue of retention is central to this project because of the length of the intervention (about 25 min) and the fact that participants did not necessarily ask for help regarding their substance use, nor started a behavior change process (65). Preliminary analysis of user characteristics reveals that the MONBUZZ.ca has reached a vulnerable population. In fact, while 90.4% of participants have risky or problematic substance use profiles, only 20.5% have already used addiction services. In addition, of those who reported having sex during the last year (83.6%), 75.4% reported that their substance use decreased their ability to develop safer sexual practices (65).

The findings suggest MONBUZZ.ca's has the ability to reach people with risky or problematic substance use and engage them in a brief online intervention. However, the effectiveness of the intervention to (1) sensitize people about substance use; (2) to guide them to the adequate services; and (3) to reduce health-related substance use sexual risks over the time remains to be proven?. Among the MSM who completed the assessments, a negligible number agreed to be contacted at two time intervals to follow the evolution in their substance use, their sexual behaviors and their experiences on the site. This situation was due to a problem with the website that could not be fixed during the research process. The few studies that evaluated online interventions with MSM substance users in sexual contexts, targeted people who have already decided to change their behaviors and were recruited offline (35). In addition, these interventions consisted of sending text messages, which did not require participants to have an ongoing interventional commitment (35). For the effect evaluation, a consultation process with experts and potential users seems necessary to identify the main effect indicators for a project like MONBUZZ.ca as well as to model those indicators in an evaluation protocol. Moreover, since we have used a participative approach of co-construction for the development of the platform, it is logical that this evaluation process follow this same approach. Thus, an evaluation component of the MONBUZZ.ca project will be developed soon.

## Ethics Statement

This project has received ethical approval from the local research ethics committee (Project number AA-HCLM-16-016) and the research committee of the associated community-based organization.

## Author Contributions

JF-A and MG are the principal investigators of the MONBUZZ.ca project. CL-O was a project intern. The manuscript was prepared by JF-A and MG. Work associated with the literature review was conducted by CL-O and JF-A. The reviews were conducted by MG and CL-O.

### Conflict of Interest Statement

The authors declare that the research was conducted in the absence of any commercial or financial relationships that could be construed as a potential conflict of interest.
